# Therapeutic Potential of BMP7 in the Treatment of Osteoporosis Caused by the Interaction between Inflammation and Corticosteroids in Inflammatory Bowel Disease

**DOI:** 10.3390/biomedicines11082161

**Published:** 2023-08-01

**Authors:** Ivana Smoljan, Dijana Detel, Suncica Buljevic, Igor Erjavec, Ivana Marić

**Affiliations:** 1 Department of Internal Medicine, Faculty of Medicine, University of Rijeka, Brace Branchetta 20, 51000 Rijeka, Croatia; ivana.smoljan@uniri.hr; 2 Department of Cardiovascular Diseases, Clinical Hospital Center Rijeka, Kresimirova 42, 51000 Rijeka, Croatia; 3 Department of Medical Chemistry, Biochemistry and Clinical Chemistry, Faculty of Medicine, University of Rijeka, Brace Branchetta 20, 51000 Rijeka, Croatia; suncica.buljevic@uniri.hr; 4 Laboratory of Mineralized Tissues, Center for Translational and Clinical Research, School of Medicine, University of Zagreb, 10000 Zagreb, Croatia; igor.erjavec@mef.hr; 5 Department of Anatomy, Faculty of Medicine, University of Rijeka, Brace Branchetta 20, 51000 Rijeka, Croatia; ivana.maric@medri.uniri.hr

**Keywords:** inflammatory bowel disease, bone morphogenetic proteins, nuclear factor-κB ligand activator, osteoprotegerin, cytokines, macrophage activation, bone microarchitecture

## Abstract

Individuals with inflammatory bowel disease (IBD) have an increased risk of bone impairment, which is a process controlled by the RANKL/RANK/OPG system, mostly due to chronic inflammation and corticosteroid treatment. Bone morphogenic protein 7 (BMP7) has a complex role in maintaining inflammation and bone remodeling but little is known about its anti-inflammatory potential in chronic colitis. We investigated the effect of systemically administered BMP7 and corticosteroids on the severity of inflammation, macrophage differentiation, and bone regeneration in a chronic IBD model. Methods: Chronic colitis was induced in male Sprague Dawley rats via weekly administration of 2,4,6-trinitrobenzenesulfonic acid over 21 days following BMP7 or corticosteroid treatment for five days. The levels of serum and colon tissue inflammatory cytokines, RANKL/OPG system, as well as markers of macrophage polarization, were detected using RT-PCR, ELISA, or immunohistochemistry. Long bone and spine analyses were performed using microcomputed tomography (micro-CT). Results: The administration of BMP7 reduced the adverse effects of colitis and led to elevated OPG and RANK in the colon with a simultaneous decrease in TNF-α and an increase in IL-10 and TGF-β. Decreased expression of the M2 macrophage marker CD163 was found in the BMP7-treated rats compared with the colitis group, whereas the number of M1 marker iNOS-positive cells did not differ between the groups. As a result of the BMP7 treatment, morphometric parameters of trabecular bone increased, and increased trabecular separation noted in the colitis group did not appear. Conclusions: We showed that BMP7 suppressed the inflammatory response in chronic colitis, mainly by shifting the cytokine balance and by triggering alterations in the RANKL/OPG system rather than through a macrophage polarization imbalance. In addition, considering the demonstrated effect of BMP7 on bone morphology and structure, it can be suggested that BMP7 plays a role in the managing of osteoporosis in chronic colitis, and thus, its therapeutic potential in the treatment of IBD should be further evaluated.

## 1. Introduction

Inflammatory bowel disease (IBD) is a chronic disease of the gastrointestinal tract with two main subtypes: Crohn’s disease (CD), in which all sections of the gastrointestinal tract can be affected, and ulcerative colitis (UC), in which inflammation is limited to the mucosa of the colon. IBD develops as a result of uncontrolled inflammation due to food components or intestinal flora in people with a genetic predisposition and typically in combination with one or more environmental factors, including mucosal barrier dysfunction, poorly controlled immune response, and general lifestyle [1]. Nowadays, it is considered a global disease, with a prevalence of more than 0.3% (and rising), which makes it a serious threat to human health [2]. CD and UC both commonly present with abdominal pain and diarrhea as the most prominent symptoms of the disease, but up to 50% of patients experience at least one extra-intestinal manifestation involving the musculoskeletal system, hepatobiliary tract, skin, and eyes [3]. Conventional treatments aim to control symptoms mostly through pharmacotherapy, although new therapeutic approaches are constantly being developed due to the large proportion of patients who either fail to respond or react poorly to the available strategies [4].

Among various aforementioned complications in IBD, osteoporosis constitutes a significant medical problem with a prevalence of 18 to 42% [5,6]. Osteoporosis is an undesirable condition because it leads to an increased risk of non-traumatic fractures, which are associated with significant morbidity and mortality and impose an additional financial burden on the healthcare system [7]. The etiology of bone loss in IBD involves several factors that can roughly be divided into a group of variables primarily associated with general skeletal health, such as low mineral intake, decreased vitamin D synthesis, and physical activity, and IBD-associated factors, including harmful effects of prolonged administration of corticosteroids and gastrointestinal damage as a result of an unceasing inflammatory process [3,8]. A typical component of conventional treatment of IBD is corticosteroids. Despite a wide range of documented adverse side effects of their use, they are still the most commonly used anti-inflammatory drugs and the first-line therapy in the treatment of IBD-induced inflammation [6,9]. Corticosteroids exert their effects on bone metabolism through a complex mechanism involving multiple signaling pathways. They can prolong the life of mature osteoclasts under certain conditions, but ultimately decrease bone remodeling by decimating the population of osteoblasts. In addition, their application leads to the disruption of calcium homeostasis and alters the structures of collagen and matrix proteins, affecting bone strength and integrity [10].

Differentiation and formation of the bone are controlled by three different proteins: nuclear factor-κB ligand activator (RANKL), nuclear factor κB receptor activator (RANK), and osteoprotegerin (OPG). OPG acts as a soluble receptor to RANKL and competes with RANK for RANKL binding, acting as an inhibitor of osteoclast genesis and bone loss. When corticosteroids are applied exogenously in high doses, they increase the expression of pro-osteoclastogenic factor RANKL and suppress the expression of OPG, which results in the suppression of osteoblast differentiation. They also inhibit the intracellular Wnt canonical pathway primarily by increasing DKK-1 expression [9]. Administration of corticosteroids also suppresses the production of cytokines involved in the recruitment of inflammatory cells to the site of the inflammation [11].

Bone morphogenetic proteins (BMPs) were discovered as major survival factors in the regeneration of the bone but nowadays are known to be involved in several other metabolic pathways, including tissue differentiation, regulation of apoptosis, and the organogenesis of both skeletal and non-skeletal tissue [12,13,14]. Furthermore, it was suggested that BMPs also have an important function in immune-mediated disorders due to their impact on the immune system, in particular in systemic chronic diseases, such as liver disease, rheumatoid arthritis, ankylosing spondylitis, and IBD [15]. BMP7, which is an important member of the transforming growth factor β (TGF-β) superfamily, is a powerful anti-inflammatory growth factor that stimulates cells by binding to its specific BMPR2 receptor, triggering the transduction of cell signals over SMAD and mitogen-activated protein kinase (MAPK)-dependent pathways. Its mechanism of action involves counterbalancing the profibrotic activity of TGF-β in different diseases and stimulating the fibrogenic response by inhibiting the epithelial-to-mesenchymal transition [16]. Several studies showed that the anti-inflammatory effect attributed to BMP7 is achieved by reducing the basal and TNFα-stimulated expression of the proinflammatory cytokines, such as IL-6, IL-8, and IL-1β, and chemokine MCP-1, as well as by enhancing M2 macrophage differentiation [17,18].

An excessive inflammatory response in IBD becomes activated upon the detection of gut injury or infection in genetically susceptible individuals. Macrophages are crucial regulators of the gut’s immune system and gastrointestinal physiology, including gut motility and secretion, but exert a dual role in the development and resolution of IBD—they work as innate immunity effector cells and contribute to the progression of intestinal inflammation. The differentiation of macrophages is commonly classified as classically activated M1 inflammatory macrophages and alternatively stimulated M2 macrophages. Different research studies showed that M2 macrophages regulate the expression of Foxp3+ Tregs and produce IL-10 and TGF-β [19]. Furthermore, when pathogens are transported across the damaged intestinal barrier, they trigger macrophages to generate cytokines, including IL-6, IL-18, TGF-β, and TNF-α, resulting in the activation of NF-κB signal transduction pathway, infiltration of neutrophils, and inhibition of T and B cell proliferation [20]. It was previously reported that BMP7 enhances M2 macrophage differentiation and anti-inflammatory cytokine secretion in vitro [21]. Also, treatment with BMP7 in an animal model of atherosclerosis resulted in the inhibition of monocyte infiltration, a decrease in pro-inflammatory cytokines and an increase in anti-inflammatory cytokines, and an increase in M2 macrophage populations [18], suggesting that the shift toward M2 differentiation could be one of the main contributions of BMP7 in inflammation. Glucocorticoids boost macrophage survival to switch off inflammation and sustain the late phase of healing but their role in the directing of M2 population in chronic colitis has not yet been investigated. As previously mentioned, despite the efficacy of corticosteroids in controlling the acute phase of IBD, they are not an ideal option for long-term treatment due to a variety of side effects [4].

Hence, the goal of this study was to investigate the interplay between RANKL/RANK/OPG, which are three factors that are important for the development of osteoporosis in IBD, and to study the effects of BMP7 and glucocorticoid treatments on their expression in the affected intestinal mucosa and serum. In addition, we wanted to test whether BMP7 treatment affects the shift in macrophage balance toward M2 subpopulation and its repercussions on bone morphology and structure as part of its anti-inflammatory action.

## 2. Materials and Methods

### 2.1. Animals

Male Sprague Dawley rats (Harlan, Germany), 4–6 weeks old, weighing 230–250 g were used and handled in accordance with institutional and national guidelines. The rats were housed in groups in standard laboratory cages in temperature-controlled conditions and fed with a standard laboratory chow diet and tap water ad libitum. The fundamental ethical principles of working with animals were respected during the experiments. All animal procedures were performed following the Croatian Law for the Protection of Laboratory Animals, which has been harmonized with the EU legislation (Council Directive 86/609/EEC, following the “Guide for the Care and Use of Laboratory Animals” published by the National Institutes of Health (NIH) and were approved by the Ethical Committee of the Faculty of Medicine, University of Rijeka (EP 87/2017), and by a national authority (Ministry of Agriculture), license HR-POK-024).

### 2.2. Chronic Colitis Induction and Assessment of Severity

**Figure 1:**
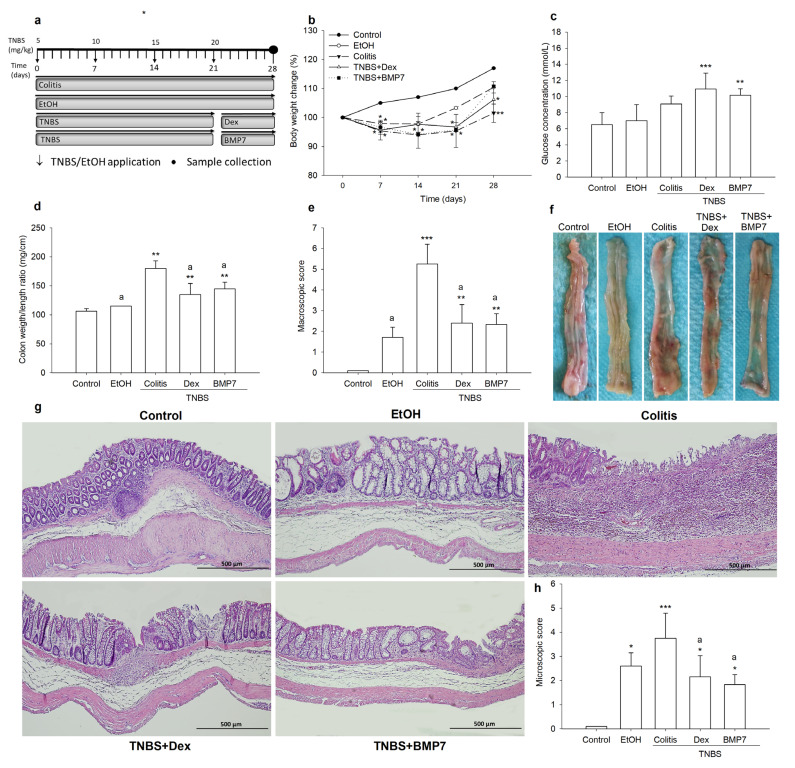
Effect of the Dex and BMP7 treatments on clinical, macroscopic, and microscopic features of TNBS-induced chronic colitis. (**a**) Schematic illustration of the experimental protocol. Chronic colitis was induced via weekly intrarectal administration of TNBS for 4 weeks (days 0, 7, 14, and 21). Rats were sacrificed on day 28. The control group received tap water. (**b**) Body weight changes were calculated as the percentage of initial weight ± SD. (**c**) Blood glucose concentration. (**d**) The weight/length ratio of the colon. (**e**) Macroscopic score calculated as described in Section 2. (**f**) Representative macroscopic images of colon specimens from all groups. (**g**) Hematoxylin and eosin images of colon tissue sections on day 28 that demonstrate microscopic changes. Original magnification: ×100. (**h**) Microscopic damage score. Student’s *t*-test was used to analyze the differences between groups. Data are presented as the mean ± SD. N = 6–8 rats/group. * *p* < 0.05, ** *p* < 0.01, *** *p* < 0.001 compared with the control group; a: *p* < 0.05 compared with the colitis group.

The chronic experimental model of IBD was induced as described previously [22]. Briefly, animals were anesthetized with an intraperitoneal injection of 100 mg/kg ketamine (Agener, Sao Paulo, Brazil) and xylazine solution (10 mg/kg, Ceva, Paulínia, Brazil). Chronic experimental colitis was induced via intracolonic administration of 2,4,6-trinitrobenzenesulfonic acid (TNBS; Sigma-Aldrich, Taufkirchen, Germany) dissolved in 50% ethanol (Kemika, Zagreb, Croatia). Each animal received 200 µL of TNBS solution using a plastic catheter inserted 8 cm proximal from the anus. The animals were kept in a head-down position for 20–35 s to avoid the leakage of the TNBS solution. Chronic colitis was induced via weekly (days 0, 7, 14, and 21) intrarectal applications of increasing concentrations of TNBS (5, 10, 15, and 20 mg/kg) [23,24]. Rats were divided into the following groups: (1) control rats receiving tap water during the experimental period; (2) EtOH group rats receiving 50% vol/vol ethanol; (3) TNBS colitis group rats receiving TNBS enema in 50% ethanol solution; and (4) and (5) TNBS-colitis-treated groups that were subjected to either BMP7 or corticosteroid treatment 24 h after the last dose of TNBS, respectively (Figure 1a). Treatments were given for 5 days according to the previously published protocols [24,25]. BMP7 (Creative BioMolecules, Inc., Hopkinton, MA, USA) was administrated intravenously (100 µg/kg), while dexamethasone (Krka, Novo Mesto, Slovenia) was administrated subcutaneously (2 mg/kg) at 1, 2, 3, and 5 days after the colitis induction. Each group included 6–8 animals. During the experimental protocol, rats were checked daily for indicators of colonic inflammation, including body mass, rectal bleeding, and diarrhea. At specified times, animals were sacrificed via cervical dislocation under isoflurane anesthesia and their colons were isolated, cleaned, and measured for weight and length, with an evaluation of macroscopic appearance. Furthermore, colon samples were processed for further analysis, including histology, immunocytochemistry, and RT-qPCR. Additionally, blood was collected, centrifuged at 15,000× *g*, and the serum was transferred to sterile tubes and stored at −80 °C until further analysis.

### 2.3. Evaluation of Colonic Damage

During the experimental protocol, the animals were weighed (days 7, 14, 21, and 28). We confirmThe distal part of the rat colon was dissected, washed, and examined for the presence of colitis-induced lesions. Furthermore, the colon was examined by two investigators in a blind fashion. Macroscopically visible colonic damage was assessed using a scoring system described previously with slight modifications [26,27] by adding individual scores for the presence of obstruction of the colon, and adhesion as defined in Table 1. The presence of diarrhea, obstruction by fecal impaction, presence of fecal blood, and proximal dilatation increased the score by 1 grade for each additional feature. Colon samples were also taken for microscopic analysis and were examined by two independent observers and scored according to the previously established criteria with slight modifications, considering several parameters including the degree of epithelial damage, infiltration with inflammatory cells, extent of the lesions, and severity of edema [28,29]. Grade 0, normal structure of the mucosa, submucosa, muscularis propria, and serosa without infiltration of inflammatory cells and edema; grade 1, surface epithelium damage with inflammatory cells in the mucosa; grade 2, mucosal erosions/ulcerations with inflammatory cells infiltration in the mucosa and submucosa, and edema in the mucosa; grade 3, focal transmural ulceration, a transmural expansion of immune cells, and edema in the mucosa and submucosa; grade 4, multiple large transmural ulcerations and inflammation, edema in the mucosa and submucosa; grade 5, extensive ulcerations involving the entire segment, transmural inflammation, and edema in entire colon wall. The remaining colon samples were stored in liquid nitrogen for additional analysis.

### 2.4. Enzyme-Linked Immunosorbent Assay (ELISA)

The serum concentration of OPG and RANKL were assayed using commercially available ELISA kits (Immundiagnostik AG, Bensheim, Germany) according to the manufacturer’s guidelines. To optimize the experiment protocol before the assay, an ELISA-based dilution factor test was run. All measurements were performed in duplicates (EL808, BioTek Instruments, Inc., Winooski, VT, USA). The data were calculated using a four-parameter logistic curve.

### 2.5. Real-Time Polymerase Chain Reaction Analysis of Gene Expression

Frozen colon segments were subjected to homogenization (Polytron PT 1600E, Kinematica AG, Luzern, Switzerland). TRI Reagent (Ambion, Austin, TX, USA) was used for the extraction of total RNA in accordance with the manufacturer’s instructions. RNA was finally dissolved in diethylpyrocarbonate water (Ambion, Austin, TX, USA) and stored at −80 °C after we quantified its content spectrophotometrically at 260 nm. Before the experiment, the integrity of the extracted RNA was checked with 2% agarose gel electrophoresis. Lastly, RNA (2 µg) was transcribed into complementary DNA with a High-Capacity cDNA Reverse Transcription Kit (Applied Biosystems, Foster City, CA, USA), along with the samples not containing reverse transcriptase for the exclusion of DNA-based contamination. The expression of the targeted genes was quantified with SYBR green die (Applied Biosystems, Foster City, CA, USA) with specific primers whose sequences are stated in Table 2.

Each reaction contained 5 µg of cDNA in a 25 µL mixture and was done in duplicate with the 7300 RT-PCR System (Applied Biosystems, Foster City, CA, USA). The 2^−ΔΔCt^ method was used for the calculation of relative changes in mRNA expression and GAPDH was used as the housekeeping gene.

### 2.6. Immunohistochemical Analysis

Immunohistochemical analysis was performed on 5 µm paraffin-embedded tissue sections as previously described [30]. Sections were deparaffinized in xylene and rehydrated, followed by heat antigen retrieval in a citrate buffer (0.01 M, pH 6.0). Sections were then incubated with 5% bovine serum albumin (BSA) and finally with primary antibody against OPG/Osteoprotegerin (1:100 dilution, ab73400, Abcam, Cambridge, UK), CD163 (mouse mA, 1:100 dilution, NCL-L-CD163, Leica Biosystems Newcastle Ltd., Newcastle upon Tyne, UK), and iNOS (1:200 dilution, ab3523, Abcam, Cambridge, UK) diluted in 1% BSA/PBS in a humidified chamber at 4 °C overnight. Staining was performed using the Dako REAL™ EnVision™ kit according to the manufacturer’s instructions, and sections were counterstained with hematoxylin, dehydrated, and cover-slipped with BioMount mounting media (Biognost, Zagreb, Croatia). The slides were visualized under an Olympus BX51 light microscope and the images were edited using an Olympus DP70 digital camera (Olympus Corporation, Tokyo, Japan). The average number of CD163 positive cells for each experimental group was determined by counting the positive cells in 10 randomly selected areas per slide for each animal in each group.

### 2.7. Bone Morphometric Analysis

The extracted left femur and lumbar spine were imaged using a Micro-CT scanner (SkyScan 1076, Bruker, Kontich, Belgium) with a 9 µm isotropic voxel size, a voltage of 50 kV, and an electric current of 200 µA. Beam-hardening was reduced using a 0.5 mm thick aluminum filter. The rotational shift was set to 0.5° in an area of 198° for every sample. After the acquisition, image reconstruction was performed using the NRecon software, version 1.7.3.1. (Bruker, Kontich, Belgium).

The acquired sample data sets were analyzed using CTAn software, version 1.19.11.1 (Bruker, Kontich, Belgium). Morphometric analysis for trabecular bone analysis was performed on the distal femur, while the midshafts of the femur were used for cortical bone analysis. For spinal bone, a vertebral body of 4 lumbar vertebrae was chosen [31,32]. Morphometric analysis of the femur was conducted using the distal growth plate as a reference point. To avoid primary spongiosa, the offset from the growth plate was 50 slices for trabecular bone, while a 350-slice offset was used for cortical bone. The volume of interest was 50 slices for cortical bone and 100 slices for trabecular bone. Trabecular bone was manually delineated from cortical bone and analyzed as such. For the vertebral body, the distal growth plate was determined as a reference point with a 50-slice offset with the following 100 slices for trabecular bone analysis. The trabecular bone was manually delineated from cortical bone and analyzed.

The following output parameters for femoral and vertebral trabecular bone were analyzed [33]: percent bone volume (BV/TV), trabecular number (Tb. N.), trabecular thickness (Tb. Th.), trabecular separation (Tb. Sb.), trabecular pattern factor (Tb. Pf.), and connectivity density (Conn. Dn.). The following parameters were analyzed for the femoral cortical bone [33]: BV/TV, endosteal volume (EV), and cortical bone surface (BS). BV/TV serves as an index for evaluating the changes in bone mass and reflects the trabecular morphological structure, while Tb. N. indicates the number of intersections between bone tissue and non-bone tissue in a given section. The trabecular separation (Tb. Sp) reflects the bone trabecular morphology and structure, as it represents the average width of the medullary cavity between bone trabeculae. Furthermore, Conn. D. is the degree of interconnection between bone trabeculae structures, which indicates the quantity of connection in the trabecular meshwork and, together with BS, is an indicator of bone loss. EV represents the volume within the medullary cavity and was determined by subtracting BV from TV.

### 2.8. Statistical Analysis

Data are shown as the mean  ±  standard deviation. Statistical analysis was conducted using STATISTICA version 12.0 (StatSoft Inc., Tulsa, OK, USA). Student’s *t*-test, the Mann–Whitney U test, or one-way ANOVA with Tukey’s post hoc test were used to evaluate differences between groups. A value of *p* < 0.05 was considered statistically significant. To establish the association between morphometric bone parameters, a Pearson’s correlation coefficient test was used (r). Linear regression was expressed as the coefficient of determination r^2^. It was used to verify the proportion of the alteration in BV/TV that was predictable from the alteration of specific morphometric bone parameters.

## 3. Results

### 3.1. BMP7 Treatment Diminished Clinical Symptoms and Reduced the Damage of Colonic Tissue

To investigate whether the BMP7 treatment affected the resolution of colitis, we used a TNBS-induced experimental model of colitis. In addition to the control group, which received water throughout the experiment, we formed a group of animals that were given only ethanol solution to exclude its effect on the investigated inflammatory mediator profiles. Given that ethanol did not trigger changes in the expression of the determined mediators, comments on that experimental group are given only when a significant change was noticed, primarily in the macro- and microanalysis of the colon.

The progression and resolution of colitis were monitored by observing clinical parameters and via pathohistological analysis. During the development of colitis, the decrease in body weight in all TNBS-treated animals was determined (Figure 1b). In both the Dex- and BMP7-treated rats, the weight gain was more pronounced than in the rats treated only with TNBS on the 28th day of the experiment. Compared with the control group, the decrease in body weight was 13.39% in the colitis group and 9.4% and 5.5% in the Dex and BMP7 groups, respectively. Although the weight gain in the BMP7 group was more pronounced compared with the Dex group (110.76 g ± 0.44 g vs. 106.14 g ± 4.13 g), no significant differences were observed between these groups. The colon weight/length ratio, which is a marker of colonic damage and tissue edema, was significantly higher in the colitis group compared with the control, Dex, and BMP7 groups (Figure 1d). Furthermore, it was decreased in the group treated with Dex and BMP7 compared with the colitis group (*p* = 0.024 and *p* = 0.04, respectively). A significant increase in the macroscopic score was observed in all TNBS-treated groups compared with the control group. However, as shown in Figure 1e, the administration of Dex and BMP7 reduced the effect of TNBS and, consequently, the macroscopic score, as well as the microscopic score (Figure 1h), decreased significantly in both groups compared with the rats treated only with TNBS (*p* < 0.01). The EtOH group had a higher macroscopic score than the control group, but it was not significant (Figure 1e). Also, the microscopic score was significantly increased in the colitis, Dex, BMP7, and EtOH groups compared with the control group (Figure 1h). Histopathologic changes in colitis were associated with extensive infiltration of inflammatory cells, deformation of crypts, depletion of cells, and submucosal edema (Figure 1g). Treatment with Dex and BMP7 ameliorated the extent of crypt distortion and loss of colonic architecture and cell infiltrations, resulting in significantly lower microscopic scores compared with rats treated with TNBS alone (Figure 1h). The demonstrated reduction in the extent of colonic damage in both the Dex and BMP7 groups indicated the anti-inflammatory effect of both treatments. Hence, we concluded that the resolution of colitis was positively affected by treatment with both Dex and BMP7.

### 3.2. BMP7 Treatment Affected the Gene Expressions and Protein Concentrations of OPG/RANKL/RANK

**Figure 2:**
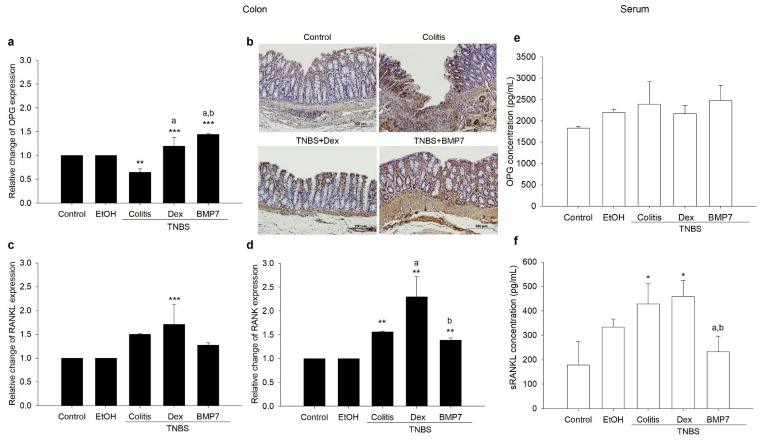
Effects of Dex and BMP7 treatments on OPG, RANKL, and RANK colonic tissue expressions and serum concentrations in TNBS-induced chronic colitis. (**a**) Gene expression of OPG in colonic tissue. (**b**) Representative OPG-stained colon sections. Original magnification: ×100. (**c**,**d**) mRNA expression of RANKL and RANK. (**e**,**f**) Serum concentrations of OPG and sRANKL determined by enzyme-linked immunosorbent assay. Data are presented as the mean ± SD. N = 6–8 rats/group. Student’s *t*-test was used to analyze the differences between groups. Original magnification: ×200. * *p* < 0.05, ** *p* < 0.01, *** *p* < 0.001 compared with the control group. a: *p* < 0.05 compared with the colitis group. b: *p* < 0.05 compared with the Dex group.

To establish the roles of OPG, RANKL, and RANK in TNBS colitis development and resolution, we investigated their gene expression in colonic tissue and their serum concentration. A decreased gene expression of OPG was found in rats treated only with TNBS in comparison with the control group, as well as the Dex and BMP7 groups (Figure 2a). Furthermore, an increase in its expression was observed in both the Dex and BMP7 groups. A similar protein expression pattern obtained by immunohistochemical staining was observed in the epithelial cells of the colon mucosa (Figure 2b).

However, in rats treated only with TNBS, the expression of OPG protein was correlated with the number of inflammatory cells (Figure 2b). The gene expression of RANKL was affected by the Dex administration, causing a significant increase. An increase was also visible in the colitis and BMP7 groups, but not compared with the control group (Figure 1c). On the other hand, RANK expression was increased in the colitis and both treated groups, but the increase was again most prominent in the Dex group (Figure 2d). As shown in Figure 2e, the serum concentration of OPG protein showed no significant changes in either group, whereas the concentration of sRANKL protein was enhanced in rats treated only with TNBS or in combination with Dex or BMP7. However, we found that in rats treated only with TNBS or in combination with Dex, the concentration of sRANKL increased significantly compared with the control group (Figure 1f). However, the BMP7 treatment annulled the previously mentioned increase, as the concentration of sRANKL was similar to the control values and decreased in comparison with colitis and Dex groups.

### 3.3. BMP7 Suppressed the Inflammatory Response in Chronic TNBS Colitis

**Figure 3:**
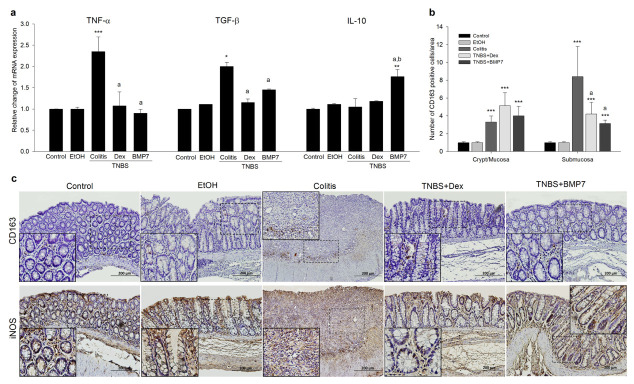
Effects of the Dex and BMP7 treatments on inflammatory disease markers expression in colonic tissue in TNBS-induced chronic colitis. (**a**) Relative mRNA expression of tumor necrosis factor α (TNF-α), transforming growth factor β (TGF-β), and interleukin 10 (IL-10), respectively. (**b**) Quantification of CD163-positive cells in the colonic mucosal and submucosal layers. The number of positive cells was calculated as the average of 10 randomly selected areas per slide per animal in each group. (**c**) Representative images of CD163- and iNOS-stained colon sections. Original magnification: ×100. The inset areas are defined by the dotted line. Student’s *t*-test, Mann–Whitney U test, or one-way ANOVA with Tukey’s post hoc test were used to evaluate the differences between groups. Data are presented as the mean ± SD. N = 6–8 rats/group. * *p* < 0.05, ** *p* < 0.01, *** *p* < 0.001 compared with the control group. a: *p* < 0.05 compared with the colitis group. b: *p* < 0.05 compared with the Dex group.

To further evaluate the role of BMP7 in the resolution of colonic inflammation during TNBS-colitis, the gene expressions of proinflammatory and anti-inflammatory cytokines were determined (Figure 3a). An opposite pattern of *TNF-α* gene expression between the rats treated only with the TNBS and rats treated with Dex and BMP7 was observed. The increase was most prominent with colitis, while no difference was observed between the Dex and BMP7 rats (1.15 ± 0.08 vs. 1.45 ± 0.05), as well as between these groups and the control (Figure 3a). Interestingly, a similar pattern was determined for the *TGF-β* gene expression, with the highest expression in rats treated only with TNBS (Figure 3a). BMP7 treatment significantly upregulated the IL-10 mRNA expression compared with all TNBS-treated rats and control. To investigate whether BMP7 therapy influenced the CD163 and iNOS protein, representative markers of M2 and M1 macrophages, as well as their protein expression and localization, were determined (Figure 3b,c). Increased CD163 protein expression was detected in the inflamed mucosal and submucosal layers, as shown in Figure 3b. In the mucosa, a significant increase in the number of CD163-positive cells was detected in all TNBS-treated groups compared with the control group, with no significant difference between the colitis, Dex, and BMP7 groups. In contrast, a significant increase in the number of CD163-positive cells in the submucosa was observed in the colitis group compared with the control, Dex, and BMP7 groups, but with no difference between the Dex- and BMP7-treated rats. On the other hand, the expression of the M1 marker iNOS was present in all specimens, both normal and inflamed, as shown in Figure 3c. In normal mucosa, superficial epithelial staining was observed, while in inflamed mucosa, additional labeling was seen in the crypt epithelium and inflammatory cells of the lamina propria. The staining was most intense in the colitis group, meaning that the increased labeling was highly likely related to the number of inflammatory cells in the lamina propria. In the Dex and BMP7 groups, iNOS was preferentially expressed in the inflammatory areas. However, we did not observe a significant difference between these groups.

### 3.4. Alterations in the Trabecular and Cortical Long Bone Parameters in Chronic Colitis

**Figure 4:**
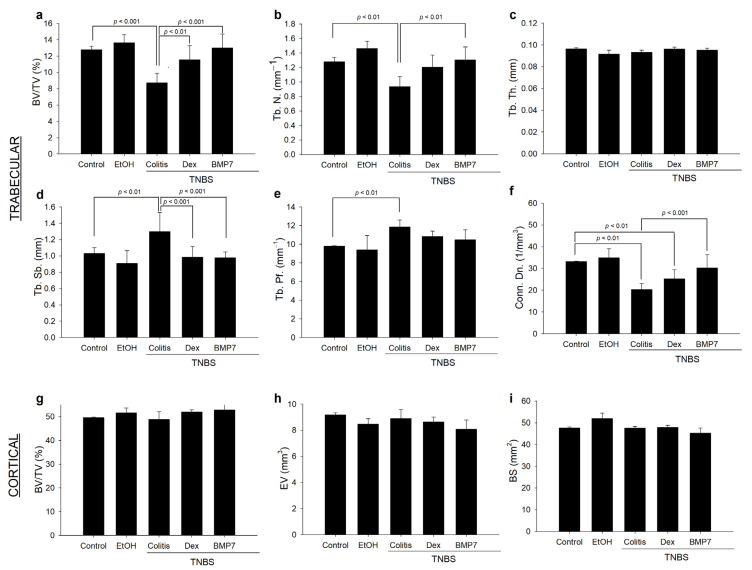
Trabecular and cortical bone morphometric parameters in the distal femur in TNBS-induced chronic colitis. Analysis of the (**a**) trabecular bone volume fraction (BV/TV), (**b**) number of trabeculae (Tb. N.), (**c**) trabecular thickness (Tb. Th.), (**d**) trabecular separation (Tb. Sp.), (**e**) trabecular pattern factor (Tb. Pf.), (**f**) trabecular connectivity density (Conn. Dn.), (**g**) cortical BV/TV, (**h**) cortical endosteal volume (EV), and (**i**) cortical bone surface (BS). Mann–Whitney U test or one-way ANOVA with Tukey’s post hoc tests were used to evaluate the differences between groups. Data are presented as the mean ± SD. N = 6–8 rats/group.

To evaluate the inflammation-related changes and the impacts of the Dex and BMP7 treatments on femur metabolism, the morphometric parameters of trabecular and cortical bone were analyzed using microcomputed tomography (micro-CT). The changes in certain morphometric parameters are shown in Figure 4. It was evidenced that the trabecular BV/TV ratio decreased by 31% in the colitis group compared with the control group. Interestingly, a significant increase was noted in the Dex and BMP7 groups compared with the colitis group (Figure 4a). The changes in trabecular number followed the trend observed in BV/TV ratio. The decrease in trabecular number was most pronounced in the colitis group, whereas significant changes between the Dex, BMP7, and control groups were not found. Interestingly, a significant increase in Tb. N. was observed in the BMP7-treated rats compared with the colitis group (Figure 4b). As shown in Figure 4c, no significant differences were observed in Tb. Th. between the experimental groups. Due to a significant decrease in the number of trabeculae, an increase in trabecular separation was observed in the colitis group, whereas this was not seen in the Dex and BMP7 groups compared with the control group (Figure 4d). While Tb. Pf. was significantly higher in the colitis group, such changes were not observed in the other experimental groups (Figure 4e). The trabecular connectivity density was decreased (*p* < 0.01) in the colitis and Dex rats compared with the controls (Figure 4f). As shown in Figure 4g–i, both colitis with and without Dex and BMP7 treatments caused no changes in morphometric parameters of cortical bone. Furthermore, analysis of trabecular and cortical spine bone revealed no significant differences between the experimental groups.

### 3.5. Serum Concentrations of OPG and sRANKL Were Inversely Correlated with Bone Morphometric Parameters

**Figure 5:**
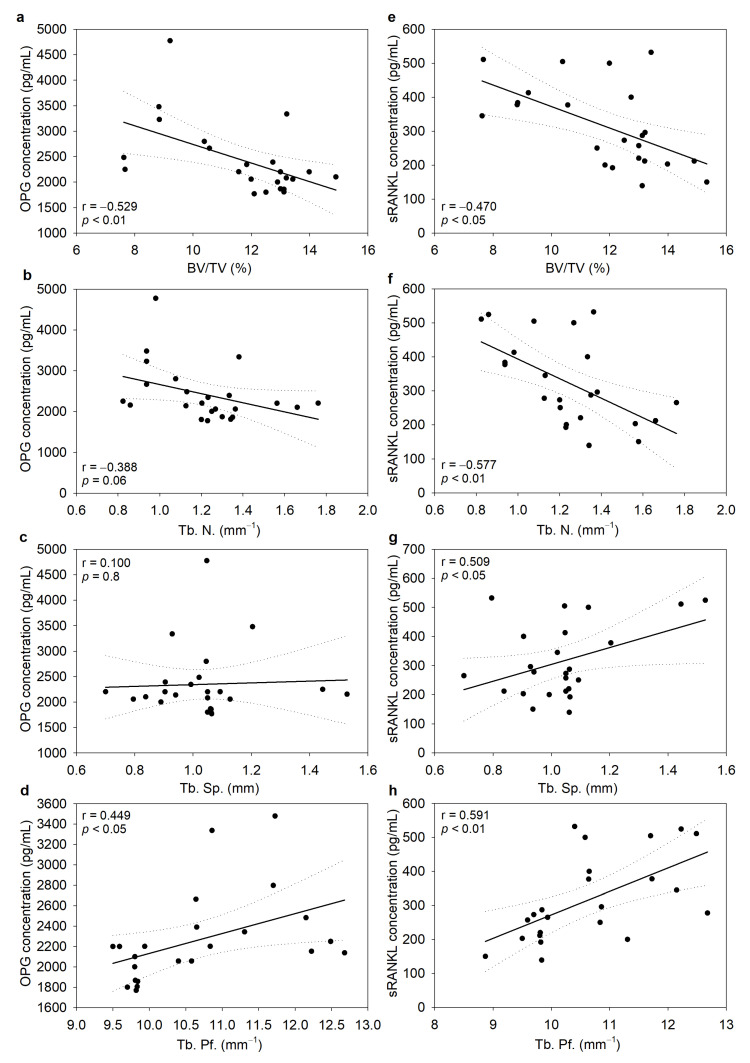
Correlations between osteoprotegerin (OPG), serum concentration of nuclear factor-κB ligand activator (sRANKL), and trabecular bone morphometric parameters of the distal femur in TNBS-induced chronic colitis. (**a**,**e**) BV/TV ratio, (**b**,**f**) number of trabeculae (Tb. N.), (**c**,**g**) trabecular separation (Tb. Sp.), and (**d**,**h**) trabecular pattern factor (Tb. Pf.). The full line represents the line of best fit, while the dotted lines represent the 95% confidence intervals. Data are expressed as the mean ± SD. N = 6–8 rats/group.

The correlation between the serum levels of OPG and RANKL with trabecular femur morphometric parameters was determined to establish the strength of an association between them. The analysis of trabecular parameters revealed a significant negative correlation between the OPG concentration and BV/TV (Figure 5a) and a positive correlation with Tb. Sp. and Tb. Pf. (Figure 5c,d). Interestingly, no such correlation was found between Tb. N. and OPG (Figure 5b). On the other hand, sRANKL correlated significantly with all studied parameters (Figure 5e–h). Compared with OPG, sRANKL showed a negative correlation with the Tb. N. values. It should be noted that although correlations between the investigated variables were found, the strengths of the linear relationship between them were considered moderate (r above 0.5) or low (r between 0.3 and 0.5).

**Figure 6:**
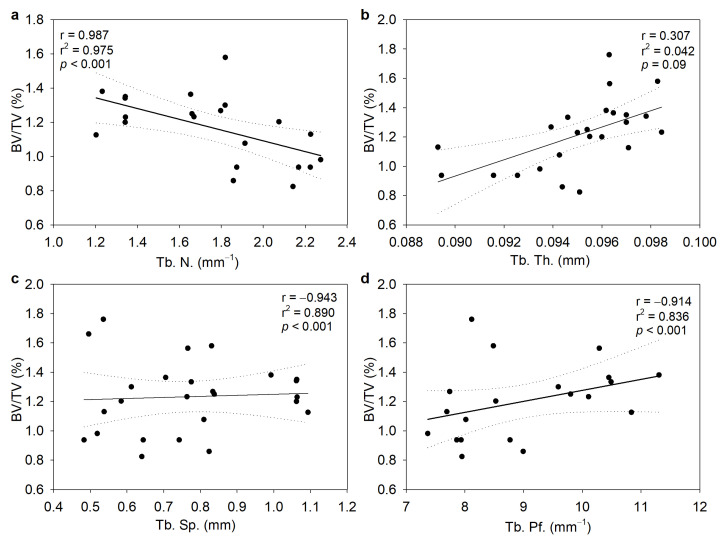
Correlation between the trabecular bone volume fraction (BV/TV) and morphometric parameters of the distal femur. (**a**) Number of trabeculae (Tb. N.), (**b**) trabecular separation (Tb. Sp.), (**c**) trabecular thickness (Tb. Th.), and (**d**) trabecular pattern factor (Tb. Pf.). The full line corresponds to the fit derived using linear regression, while the dotted lines denote the 95% confidence intervals. Data are expressed as the mean ± SD. N = 6–8 rats/group.

Correlation analysis was used to determine the association between BV/TV and other morphometric parameters, while simple linear regression was used to test their relationships. A high positive correlation between BV/TV and Tb. N. (r = 0.98) was established (Figure 6a). In addition, there was a high negative correlation between BV/TV and Tb. Sp. (r = −0.943) and Tb. Pf. (r = −0.914), whereases Tb. Th. did not change significantly (Figure 6b–d).

## 4. Discussion

According to our results, the administration of both Dex and BMP7 increased the expression of OPG in the colon, while its expression in the colitis group decreased. At the same time, the gene expression of colon RANK was increased in all three colitis groups, and RANKL expression was increased only under the Dex treatment. Dex treatment increased OPG, the process being a part of the anti-inflammatory glucocorticoid action, but also RANK expression at the site of inflammation, which could act as a counterbalance to the increase in OPG. In addition, we found that neither Dex nor BMP7 affected the plasma concentrations of OPG. On the other hand, sRANKL increased with the Dex treatment, while BMP7 did not trigger the aforementioned increase. Although increased release of OPG from the damaged colon and its high plasma levels were previously shown, our results do not support this conclusion. The authors themselves stated that the contribution of OPG and sRANKL from the mucosa to the determined serum levels was difficult to estimate in that particular study [34]. The observed increase in colon OPG, which was also noted in a mouse colitis model, could represent a sustained homeostatic response attempting to reverse existing osteopenia and RANKL-induced osteoclastogenesis to maintain normal bone mass [35]. The importance of the activated RANKL/OPG signaling pathway was previously described in chronic inflammatory disorders, including rheumatoid arthritis, with elevated levels of both sRANKL and OPG [34]. Despite their widespread use, it is, therefore, still uncertain whether the observed effects of glucocorticoid administration are due to the drug itself or represent a marker of a more severe chronic inflammatory state.

The available literature on BMP7 in inflammation is scarce, but it is known that it acts as an efficient inhibitor of apoptosis and fibrosis, exerting its anti-inflammatory properties. It was demonstrated that BMP7 binds to its BMPR-II receptor, which triggers both Smad-dependent and -independent PI3K signaling pathways with activation of the mammalian target of the rapamycin (mTOR) pathway [36]. The essential factor in immune regulation is the PI3K signaling pathway since it plays a significant role in maintaining anti-inflammatory conditions. It was shown that the Smad–PI3K–Akt–mTOR pathway specifically inhibits the secretion and production of pro-inflammatory cytokines and enhances anti-inflammatory cytokines secretion. TNF-α is considered to be the main promoter of bone loss in patients with IBD. It induces osteoclast differentiation via both RANKL-dependent and -independent signaling pathways [37]. The results of our study demonstrated that TNF-α was strongly expressed in the colon tissue affected with inflammation. Treatment with Dex decreased its expression to the level of healthy controls, and treatment with BMP7 suppressed it even more, which was consistent with our previous work that indicated a strong anti-inflammatory effect of BMP7 in colitis [38]. Anti-inflammatory cytokines IL-10 and TGF-β are known to counteract the hyperactive immune response in IBD [39,40]. We determined increased expression of these cytokines in colon samples of the chronic colitis group. Treatment with both Dex and BMP7 initiated a trend toward normalization of TGF-β expression, which was changed upon TNBS-colitis establishment, in line with our earlier study highlighting the important role of BMP7 in maintaining normal TGF-β expression in colitis [28]. The IL-10 expression remained elevated in both treatment groups, which supports the anti-inflammatory effect of both Dex and BMP7 in IBD. In addition to a potent anti-inflammatory effect, IL-10 plays an important role in antigen presentation and immune stimulation by upregulating MHC class II expression on B lymphocytes and inducing differentiation of cytotoxic T cells [39]. These multiple pleiotropic functions may additionally explain the increased IL-10 levels in treated colon samples.

Another aspect of BMP7′s role in the immune regulation of chronic inflammation is its key role in M2 macrophage polarization [21,36]. Knowledge about BMPs, especially the less investigated BMP7, in macrophage polarization is new and growing, and several unanswered questions still exist. It was shown that recombinant human BMP7 inhibits atherosclerosis-associated inflammation [41]. The observed phenomenon was explained by BMP7′s ability to promote the differentiation of monocyte into the M2 phenotype via the reduction of phosphorylated kinases p-38 and JNK with a simultaneous increase in p-Smad and ERK signaling pathways [18,41]. Furthermore, the same research group demonstrated a significant increase in BMPR-II receptor expression on monocytes following BMP7 treatment and further polarization into M2 macrophages [21]. Although we detected increased expression of the M2 marker CD163 in the inflamed mucosa and submucosa of colonic samples in the colitis, Dex, and BMP7 groups, the number of CD163-positive cells was markedly lower in the BMP7 group than in the colitis group, implying that BMP7 did not significantly direct the differentiation of macrophages into the alternatively activated M2 population. The observed effect could shed new light on the nature of the mechanisms triggered by BMP7. However, we would like to point out that a limitation of the study is that we only examined the expression of two macrophage markers so additional M1/M2 markers (i.e., CD80, CD86, Fizz, and Arg-1) should be determined to corroborate our conclusion regarding the M2 polarization. According to our results, the main source of the observed protective BMP7 effect was generated through the shift in the balance of pro- and anti-inflammatory cytokines TNF-α and IL-10 expression, with the latter not being primarily produced by macrophages but rather by other immune cells. IL-10 as a common cytokine that controls the severity of inflammation is associated with several autoimmune diseases both in humans and mice and is highly important for IBD pathogenesis since both IL-10^−/−^ and IL-10Rβ^−/−^ mice develop spontaneous enterocolitis [42]. In vivo, besides macrophages, major sources of IL-10 are T helper cells, dendritic cells, monocytes, and multiple immune effector cell types in the inflamed tissue. Additionally, epithelial cells can produce IL-10 in response to tissue damage or infection [43]. Given the observed phenomenon, we can speculate that in the TNBS-induced chronic colitis model we used, all or some of the abovementioned cells are sources of elevated IL-10 rather than M2 macrophages.

Because of the aforementioned function of corticosteroids and BMPs in bone remodeling, we compared the effects of colitis, Dex, and BMP7 on bone morphometric parameters, providing a more profound overview of the effect of BMP7 on bone remodeling. To date, there is insufficient information on the influence of BMP7 on the long bones, in particular the femur, in chronic colitis. The decreased BV/TV ratio and Tb. N. and increased Tb. Sb. and Tb. Pf. in colitis confirmed previous studies indicating that inflammation increases bone resorption [44]. In addition to the shown anti-inflammatory effect, the impact of BMP7 treatment on bone remodeling was also shown. Based on our and previously published results, a dual effect of BMP7 can be proposed. First, these effects were indirectly mediated via a demonstrated decrease in the RANKL/OPG ratio, which negatively affected osteoclast synthesis and function. Second, we showed that BMP7 treatment suppresses the expression of TNF-alpha, which is the main promoter of bone loss in patients with IBD, suggesting that these effects were the repercussion of the established anti-inflammatory role of BMP7 [28]. Although it was shown that the Dex application also increased bone resorption, the results obtained via micro-CT did not confirm this.

In summary, we demonstrated elevated OPG and RANK colon expression and decreased TNF-α and TGF-β in BMP7-treated animals, confirming the beneficial effect of BMP7 in IBD, which is, at least partially, carried out through modifications in the beforementioned pathway. Furthermore, we propose that contrary to some of the previously published data, BMP7 does not cause a switch in the M1/M2 ratio toward the M2 phenotype, implying that other immune cells are the source of the elevated IL-10 noted in the BMP7-treated group. Considering the determined effect of BMP7 treatment on the RANKL/RANK/OPG system, trabecular number, and separation, it can be suggested that BMP7 affects long bone morphology, density, and structure and, therefore, could have an impact on the managing osteoporosis in chronic colitis. Based on the results, it is still not clear whether the observed effects were the result of amelioration of inflammation, changes in the RANKL/OPG system, or the direct influence of BMP7 on bone metabolism; therefore, this issue may be one of the future research directions. Further studies should be conducted to deepen our understanding of BMP7 and its interplay with the RANKL/RANK/OPG pathway as key components of bone metabolism and to explore its therapeutic possibilities in IBD.

## Figures and Tables

**Table 1:** Macroscopic grading of colonic lesions.

Criteria	Grade
**Diarrhea**	
No	0
Yes, with or without blood	1
**Hyperemia/Ulceration**	
No hyperemia (mucosa was similar to uninflamed intestine)	0
Localized hyperemia (mild and intense) without ulcers	1
Extended hyperemia or hemorrhage without ulcers	2
Ulceration with hyperemia or bowel wall thickening (1–2 mm)	3
Ulcerations at two or more sites with or without bowel wall thickening > 2 mm	4
Major sites of damage extended > 1 cm along the colon length	5
If the damage extends > 2 cm along the colon length, the score was increased by 1 grade for each additional centimeter	6–10
**Adhesion**	
No adhesions	0
One adhesion without adhesion to other tissues or organs (colon could be separated from other tissues)	1
Two or more adhesions or adhesion to tissue or an organ	2

**Table 2:** Forward and reverse list of primers used for real-time quantitative PCR (RT-PCR) analysis.

Gene	Forward Primer	Reverse Primer
*OPG*	5′TGTGGAATAGATGTCACCCTGTGC3′	5′CACAGAGGTCAATGTCTTGGATGATC3′
*RANKL*	5′GCTTCTCAGTTCCAGCTATGTT3′	5′CGTTGCTTAACGTCATGTTAGAGATCT3′
*RANK*	5′GTCTCATCGTCCTGCTCCTCTT′	5′CAGCGTTTTCCCTCCCTTC′
*TNF-α*	5′ACTCCCAGAAAAGCAAGCAA3′	5′TGGAAGACTCCTCCCAGGTA3′
*TGF-β*	5′CTCAACACCTGCACAGCTCC3′	5′ACGATCATGTTGGACAACTGCT3′
*IL-10*	5′CTTACTGGCTGGAGTGAAGACC3′	5′AATCATTCTTCACCTGCTCC3′
*GAPDH*	5′GGACCAGGTTGTCTCCTGTG3′	5′CATTGAGAGCAATGCCAGC3′

## Data Availability

The data of this study are available from the corresponding author upon reasonable request.
